# On the Relationship between Speech Intelligibility and Fluency Indicators among English-Speaking Individuals with Parkinson's Diseases

**DOI:** 10.1155/2022/1224680

**Published:** 2022-10-03

**Authors:** Chin-Ting Liu, Shiao-Wei Chu, Yuan-Shan Chen

**Affiliations:** ^1^Department of Applied English, National Chin-Yi University of Technology, Taichung, Taiwan; ^2^Mount Dawu College, National Pingtung University, Pingtung, Taiwan

## Abstract

The purpose of the study is to investigate how much of variance in Parkinson's Disease (PD) individuals' speech intelligibility could be predicted by seven speech fluency indicators (i.e., *repetition*, *omission*, *distortion*, *correction*, *unfilled pauses*, *filled pauses,* and *speaking rate*). Speech data were retrieved from a database containing a reading task produced by a group of 16 English-speaking individuals with PD (Jaeger, Trivedi & Stadtchnitzer, 2019). The results from a multiple regression indicated that an addition of 54% of variance in the speech intelligibility scores among individuals with PD could be accounted for after the speakers' PD severity level measured based on Hoehn and Yahr's (1967) disease stage was included as a covariate. In addition, *omission* and *correction* were the two fluency indicators that contributed to the general intelligibility score in a statistically significant way. Specifically, for every one-unit gain in the number of *correction* and *omission*, speech intelligibility scores would decline by 0.687 and 0.131 point (out of a 7-point scale), respectively. The current study hence supported Magee, Copland, and Vogel's (2019) view that the language production abilities and quantified dysarthria measures among individuals with PD should be explored together. Additionally, the clinical implications based on the current findings were discussed.

## 1. Introduction

According to research on population-based studies, the prevalence estimates of individuals with Parkinson's Disease (PD) range from 139 to 961 per 100,000 people, depending on the countries under investigation and the methods employed for calculating the estimates [1–5]. As PD is characterized by the progressive death of dopaminergic neurons in the substantia nigra pars compacta [6, 7], patients with PD suffer from akinesia, bradykinesia, and rigidity of the laryngeal muscles [8–10], and these symptoms in turn affect their speech performances [7, 11]. In fact, more than 90% of individuals with PD experience dysarthrias [12, 13]; the speech difficulties resulting from neurological impairment, and which give rise to their poor speech intelligibility [14, 15]. In addition, empirical studies have suggested that speech changes might be essential early indications of PD [16–18]. Therefore, in view of the high prevalence rate of PD and the impact the disease exerts on the patients' speech communication capability, various studies have investigated the differences between the productions from a group of individuals with PD and typically-developing (TD) counterparts [19–24]. Specifically, the focuses were generally on the fluency indicators such as speaking rates, number of filled/unfilled pauses, and the frequency of speech errors.

Speech rates are usually measured based on the words or syllables produced by a speaker within a given time (e.g., words per 60 seconds) or a given word count (e.g., syllables per 100 words) [25, 26]. The term “speaking rate” is specifically used to refer to the speech rate measurement without removing the silent intervals from the sample [27]. It has been proposed that differences in speaking rates reflect a speaker's ability in speech movement planning [22]. Although many studies focusing on PD speech have included speaking rates as one of the measurements, the resulting patterns have been quite divergent and at least three views have been proposed. The first view was proposed by Metter and Hanson [23], who compared the speaking rates between seven individuals with PD and 10 age-matched TD controls in a read aloud task. These authors found high variability in speaking rates among the individuals with PD. In particular, while some PD speakers had slower speaking rates than the TD controls, the other PD speakers had faster speaking rates than the TD counterparts. The second view was proposed by Ludlow et al. [22]. In the study, 12 individuals with PD and 12 age- and sex-matched TD controls orally repeated four sentences with either a fast or a regular speaking rate after listening to the demonstration from a prerecorded stimuli tape. The results indicated that the individuals with PD demonstrated a tendency toward slower production. Finally, studies conducted by Huber and Darling [21] and Alvar, Lee, and Huber [19] showed that the speaking rates between PD and TD individuals were similar. Huber and Darling [21] invited 14 individuals with PD and 14 age- and gender-matched TD controls to read a passage. Results from the statistical analysis indicated that the two groups of participants produced similar numbers of syllables per second and the authors concluded that there was no difference in speaking rates between these two groups in reading. Alvar et al. [19] measured the duration of utterances and the number of words produced by 15 individuals with PD and 18 age-matched TD controls in a story retelling task. The results demonstrated that the two groups produced similar utterance durations and numbers of words per story retell, indicating that the speaking rates of the two groups were similar. In short, the findings pertaining to the speaking rates of individuals with PD were conflicting. Although many studies have reported that the speaking rates between PD and TD individuals were similar [19, 21], some studies showed that individuals with PD spoke slower [22] and with higher speaking rate variability [23]. As it will be discussed later in the section, the differences in the severity levels of individuals with PD among different studies might be one potential cause for the inconsistencies found in different studies.

Pause patterns in speech are another fluency indicator that is reported in studies focusing on PD speech. Pauses can be divided into two types: unfilled or filled pauses [28, 29]. An unfilled pause refers to a period of silence found in speech. A filled pause, on the other hand, occupies the period of silence by uttering a vowel-like sound (e.g., *uh*, *um*, or *er*) or an editing expression (e.g., *well*, *you know*, or *I mean*) [28, 30, 31]. The appearance of filled and unfilled pauses indicates that speakers are facing language formulation difficulties and are planning what to say next [32–35]. In terms of the filled pauses, the nature of the task might be an essential factor. Some studies found that when the task was reading a passage, the PD and TD individuals produced similar numbers of filled pauses [21]. On the other hand, when the participants were invited to speak spontaneously, the individuals with PD generally produced a higher number of filled pauses than did their TD counterparts [19, 21]. Conversely, the unfilled pause patterns from individuals with PD reported in the literature were less consistent. While some studies found that the silent pause durations or frequencies between PD and TD individuals were similar [19, 20, 22], other studies reported that individuals with PD generally had higher percentages or durations of silent pauses [19, 23]. In short, similar to speaking rates, inconsistencies could also be identified in the resulting pause patterns reported in PD speech-related literature.

Finally, the number of speech errors from individuals with PD has been consistently reported to be higher than that from TD controls [21, 24]. Walsh and Smith [24] compared the number of speech errors (e.g., omissions, false starts, and distortions) produced by 16 PD and 16 age- and gender-matched TD controls. The results indicated that the clinical group produced significantly more speech errors than did the TD controls among the six sentences included in the reading task. The authors claimed that the disrupted speech production among individuals with PD was a result of the lesions to the basal ganglia, a region that is involved in motor programming at the production level. That is, the inability for individuals with PD to smoothly transform the abstract linguistic codes into interpretable movement commands for the motor system is caused by the deficiency in basal ganglia (c.f., Miller and Guenther [36] for a model on the basal ganglia involvement in speech motor programming). Similarly, Huber and Darling [21] compared the numbers of formulation errors (e.g., repeated phrases, revised utterances, and abandoned utterances) produced by 14 individuals with PD and 14 age- and gender-matched TD controls in spontaneous speech and in reading. The results indicated that the clinical group produced more formulation errors than did the control counterparts, showing that the individuals with PD had greater difficulty in language formulation and production. Although the finding that individuals with PD produced more speech errors is well-supported in the literature, the exact types of errors were not recorded. That is, the label *speech errors* was used as an umbrella term for repetition, corrections, omissions and/or distortions. However, each subtype of speech errors might reflect different communicative functions and issues in speech production. For instance, in a reaction time experiment reported by Fox Tree [37], the participants were required to press a button as soon as they heard the key word in a carrier sentence. The results indicated that the reaction time was longer when there was a false start (a type of correction) preceding the key word; however, the reaction time was shorter when there was a repetition preceding the key word. Therefore, Fox Tree [37] concluded that false starts (a type of correction) hindered comprehension while repetitions speeded comprehension. Therefore, it is essential to understand the relationship between speech production and different subtypes of speech errors among individuals with PD.

Although many studies have explored the differences in fluency indicators between PD and TD individuals, for the following reasons, some cautions must be born in mind. First, it has been evident that the reported patterns of speaking rates and unfilled pauses were generally inconsistent. These inconsistencies might result from the differences of the PD severity levels (measured by Hoehn and Yahr's [9] disease stages) experienced by individuals who were included in different studies (c.f., [11, 20] for a discussion). Therefore, it is crucial to include PD severity level as a covariate in the analysis. Second, previous studies predominantly focused on the fluency indicator differences between groups of PD and TD individuals; however, little is known about how each fluency indicator might exert influences on the speech intelligibility of individuals with PD. This fact explains why Magee et al. [11] p. (1197) urged future researchers to combine “investigations of language production abilities and objectively quantified dysarthria measures” in future studies.

Therefore, the purpose of the current study is to investigate the contributions of each of the fluency indicators on a group of English-speaking individuals with PD by including the PD severity levels as a covariate. The specific fluency indicators that are explored in the current study include *repetition*, *omission*, *distortion*, *correction*, *unfilled pauses*, *filled pauses,* and *speaking rate*. By probing into this issue, the variation of the speech intelligibility among individuals with PD could be explained and predicted. In addition, the unique contribution of the fluency indicator subtypes, along with the strength and direction, could be identified. It is believed that the results of the study would provide essential insights in language rehabilitation as well as the progress assessments for individuals with PD.

## 2. Materials and Methods

### 2.1. Speech Samples

The speech samples included in the analyses were retrieved from the corpus *Mobile Device Voice Recordings at King's College London (MDVR-KCL) from both early and advanced Parkinson's disease patients and healthy controls* collected by Jaeger, Trivedi, and Stadtschnitzer [38]. The data were recorded by using Motorola Moto G4 Smartphones with the sampling rate of 44.1 kHz at King's College London Hospital. The recordings contained spontaneous and recitation speech productions from PD and TD participants. The data included in the current analysis were the sound files that were recorded when the 16 English-speaking individuals with PD were individually reading the North Wind and the Sun passage, as shown in (1). The demographics of the participants are shown in Table 1.

1. The orthographic version of the story *North Wind and the Sun* (adopted from [38])
2. The North Wind and the Sun were disputing which was the stronger, when a traveler came along wrapped in a warm cloak. They agreed that the one who first succeeded in making the traveler take his cloak off should be considered stronger than the other. Then the North Wind blew as hard as he could, but the more he blew the more closely did the traveler fold his cloak around him; and at last the North Wind gave up the attempt. Then the Sun shone out warmly, and immediately the traveler took off his cloak. And so the North Wind was obliged to confess that the Sun was the stronger of the two.

### 2.2. Data Coding

Beside the PD severity levels reflected by Hoehn and Yahr's [9] disease stages, an addition of eight speech variables were measured, including *speech intelligibility* and seven fluency indicators (i.e., *repetition*, *omission*, *distortion*, *correction*, *unfilled pauses*, *filled pauses*, and *speaking rate*). Speech intelligibility was measured by using a 7-point Likert scale where a score of 1 and 7 represented *completely unintelligible* and *completely intelligible*, respectively. Two trained adult native speakers of English, who were naïve to the purpose of the study, individually provided the speech intelligibility score for each of the 16 individuals with PD based on the first two sentences in (1). They were told to score the heard speech as *completely intelligible* (i.e., a score of 7) when they could comprehend the contents without extra efforts. They were suggested to give a score of 1 (i.e., *completely unintelligible*) if they could barely comprehend the speech contents even with extra efforts. After that, two exemplar speech samples were provided to the two listeners and they were explicitly told that one of the samples was an example of *completely intelligible* while the other one was an instance of *completely unintelligible*. The two listeners could only listen to the speech production from the same individual once. A Pearson product-moment correlation was conducted to evaluate the interrater reliability between the two listeners. The results showed that there was a strong correlation between the speech intelligibility scores assigned by the two listeners (*r* = .910, *n* = 16, *p* < .001). Therefore, the average speech intelligibility scores from the two listeners were used.

The procedure of word-for-word transcription was adapted from Huber and Darling [21] and Alvar et al. [19]. Precisely, a trained senior undergraduate student acted as the main transcriber and orthographically transcribed the sentences produced by each individual with PD based on the text shown in (1). The transcriber used predetermined arbitrary symbols to mark the presence of the seven fluency indicators (i.e., *repetition*, *omission*, *distortion*, *correction*, *unfilled pauses*, *filled pauses*, and *speaking rate*) based on the criteria in (2). A second transcriber checked the transcriptions as well as symbols indicating the presence of the fluency indicators to ensure accuracy. When there were any discrepancies, the two transcribers discussed them and reached a consensus. *Repetition*, *omission*, *distortion*, *correction*, *unfilled pause*, and *filled pause* were scored based on the number of the occurrences found in each of the individuals with PD. Following the definition of Robb and Gillon [27], speaking rate was scored based on the number of words produced in a second without removing the silent intervals from the sample.

(2) Criteria used for coding fluency indicators
  - Repetition: repetition of the same sound, word, phrase or clause without any modification
  - Omission: the omitted words in the production (c.f., (1))
  - Distortion: the words whose sounds and pronunciations deviated from the expected ones
  - Correction: reformulations of the previously uttered contents with modifications
  - Unfilled pause: a period of silence for or longer than 150 milliseconds [19]
  - Filled pause: a period of silence filled by uttering a vowel-like sound (e.g., *uh*, *um*, or *er*) or an editing expression (e.g., *well*, *you know*, or *I mean*)
  - Speaking rate: the number of words produced in a second without removing the silent intervals from the sample (c.f., [27])

### 2.3. Statistical Analysis

A multiple regression was used to predict the speech intelligibility of individuals with PD from the seven fluency indicators listed in (2) while including the PD severity level (as measured by Hoehn and Yahr's disease stage in Table 1) as the covariate. Specifically, the inclusion of the PD severity level could reveal how much of the variance found in speech intelligibility could be accounted for by the fluency indicators in (2) after the differences in the severity levels among those individuals with PD were taken into consideration.

## 3. Results

The descriptive statistics of the speech intelligibility scores and the fluency indicator frequencies among individuals with PD, including the mean, the standard deviation, and the score for each parameter of each speaker, are shown in Table 2. A multiple regression, including the severity level as the covariate, was performed to examine how much of the variance in the intelligibility scores among individuals with PD could be accounted for based on the fluency indicators in Table 2. The model summary of the multiple regression is shown in Table 3. The results indicated that after controlling for the severity level in the first model, the addition of the seven fluency indicators in the second model could explain the additional 54% variance in speech intelligibility (c.f., *R^2^* change) and the change in *R^2^* is statistically significant (*p* = .006). Additionally, the eight variables in the second model statistically significantly predicted the speech intelligibility scores among individuals with PD, *F* (8, 7) = 12.516, *p* = .002).

Table 4 shows the relevant contribution of each variable in the second model. It was found that *correction* and *omission* significantly predicted the speech intelligibility scores among individuals with PD. In particular, the fluency indicator *correction* accounted for 14.36% (i.e., the square of the standardized *β* -.379) of the variance in the speech intelligibility score, and one unit increase in *correction* would lead to a decrease of 0.687 in the speech intelligibility score. Additionally, the fluency indicator *omission* accounted for 60.53% (i.e., the square of the standardized *β* -.778) of the variance in the speech intelligibility score, and one unit increase in *omission* would lead to a decrease of 0.131 in the speech intelligibility score.

## 4. Discussion

The purpose of the current study was to examine the relevant contributions of each fluency indicator (i.e., *repetition*, *omission*, *distortion*, *correction*, *unfilled pauses*, *filled pauses*, and *speaking rate*) to the speech intelligibility of a group of 16 English-speaking individuals with PD while the participants' severity level was properly controlled. The results from a multiple regression showed that, by including the severity level of individuals with PD as a covariate, the addition of the seven fluency indicators could explain a large portion of the variance in the speech intelligibility scores. Furthermore, *correction* and *omission* were the two fluency indicators that significantly predicted the speech intelligibility scores. Specifically, increases in the number of *correction* and *omission* would result in decreases in the speech intelligibility scores. Based on the obtained results, several significant issues are highlighted and discussed below.

The current study supported Magee et al. [11] view that the language production abilities and quantified dysarthria measures among individuals with PD should be explored together. For instance, although individuals with PD have been reported to have slower speech rates (e.g., [22]), higher percentages or durations of unfilled pauses (e.g., [19, 23]) and more speech errors (e.g., [21, 24]) in comparison to a group of TD controls, it is still unclear how those fluency indicators contribute to the quantified dysarthria measure such as speech intelligibility. Therefore, with the inclusion of quantified dysarthria measures such as speech intelligibility scores in the current study, the exact contributions of the fluency indicators to the clarity of the speech production could be identified. This does not imply that the comparisons between TD and PD individuals' production of fluency indicators are not meaningful. In fact, with those comparisons, the impact of PD on the patients' speech motor abilities could be revealed. However, the understanding of the factors contributing to the speech clarity of individuals with PD could be further identified once quantified dysarthria measures and language production abilities are explored together.

Previous studies used the semantically more general term *errors* to refer to at least four types of speech errors, including *repetition*, *corrections*, *omissions*, and *distortions* [21, 24]. However, it is necessary to decompose the term *errors* into different subtypes when one wishes to evaluate the speech of individuals with PD. According to the findings, the two most influential fluency indicators that exerted statistically significant negative effects on the speech intelligibility scores among individuals with PD were the numbers of *correction* and *omission* in their reading speech productions. Of these two fluency indicators, *correction* might be a more predominant factor because one unit increase in the number of *correction* would lead to a larger decrease in the speech intelligibility score. To be specific, the speech intelligibility was measured by using a 7-point Likert scale, which effectively means that one unit increase in the number of *correction* would lead to nearly a 10% decrease in the overall speech intelligibility. Similarly, one instance increase in *omission* would lead to nearly a 2% decrease in the speech intelligibility score. Therefore, different subcategories weigh differently in speech intelligibility variation and thus future studies are suggested to decompose *errors* into distinct smaller meaningful units such as *repetition*, *corrections*, *omissions*, and *distortions*.

One straightforward clinical implication from the current study is that assessment and intervention pertaining to *correction* and *omission* are suggested to be prioritized in clinical and therapeutic sessions. This does not suggest that these two fluency indicators should be the *only* focuses. However, if the purpose of the clinical sessions is to increase the speech intelligibility of the dysarthric individuals with PD and in turn to improve their life quality, targeting *correction* and *omission* might be of great help. For instance, Miller et al. [39] focused on how changes in communication impacted the quality of life among a group of 37 individuals with PD in a qualitative study. The interviews revealed that *omission* (e.g., “It's hard work trying to talk, trying to get the words out” ([39], p. 236)) and need for *correction* (e.g., “I want to say something but something different comes out …” ([39], p. 236)) were indeed two of the communication issues individuals with PD were aware of. The authors also proposed that delayed referral to speech–language therapy for intelligibility intervention would lead the PD patient to avoid full socialization. Therefore, in order to minimize the occurrences of *correction* and *omission* in speech, specific speech activities and strategies could be designed to help those in need to properly recite the words in the first place (instead of revisiting and reformulating the previously uttered contents) and to faithfully recite the incoming written words (instead of skipping the words). Behavioural approaches such as verbal reinforcement, metronome pacing, and pacing boards (c.f., [40]) as well as instructing to use conversational repair strategies (c.f., [41]) might be appropriate in this case. It is believed that endeavour in this direction would truly help the speech intelligibility of individuals with PD.

There were certain limitations in the current study that might provide some directions for future research. First, the current study analysed data from native speakers of English. Therefore, it is unclear if the relationship between speech intelligibility and fluency indicators found in the study was a language-specific or a universal phenomenon. Cross-linguistic analysis might be particularly helpful in this respect. Take Kim and Choi's [42] study for instance. In the study, the authors explored how much variance in the speech intelligibility scores could be accounted for by four acoustic parameters among a group of English and a group of Korean speakers with PD. The results indicated that *vowel space* was an effective predictor for both language speakers with PD. However, *voice onset time* and *articulation rate* were two additional significant variables that explained variance in the speech intelligibility scores only among Korean speakers with PD. The study from Kim and Choi [42] clearly indicated that some of the factors that influenced speech intelligibility might be language universal (e.g., *vowel space*), while the others might be language-specific (e.g., *voice onset time* and *articulation rate).* Therefore, with additional investigations into the relationship between speech intelligibility and fluency indicators among different language speakers with PD, the language-specific and the language universal fluency indicators that could explain variance in speech intelligibility among individuals with PD could be unveiled. Second, a number of studies have identified that the quality of consonantal productions contribute to speech intelligibility among TD children [43] and dysarthric individuals [44–46]. For instance, both Ansel and Kent's [44] and Liu and Chen's [46] studies focused on English-speaking dysarthric individuals secondary to cerebral palsy (CP). It was found that fricative-affricative rise time contrast [44] and the numbers of vocal folds' free vibration [46] significantly contributed to the speech intelligibility of those dysarthric individuals. As consonantal productions were effective predictors of the speech intelligibility among dysarthric individuals secondary to CP, quantifiable measures of consonants are suggested to be included to further investigate how much of variance in speech intelligibility could be attributed to the quality of consonantal productions among dysarthric individuals with PD. Finally, the data in the current study were based on a reading task. However, as Huber and Darling [21] have shown that the speech behaviours in the spontaneous speech productions and reading tasks were different among the same group of individuals with PD, it would be desirable to examine if the patterns found in the current study would be similar or different from those found in a spontaneous speech production task. It is believed that future inquiries in these directions would contribute much to our understanding of the nature of the speech quality among individuals with PD.

## Figures and Tables

**Table 1:** The demographics of the PD participants included in the analysis.

Number	Hoehn and Yahr's disease Stage^b^	UPDRS^a^ II-5^b^ (activities of daily living-speech)	UPDRS^a^ III-18^b^ (motor examination-speech)
1.	2	0	0
2.	2	0	1
3.	3	1	1
4.	2	0	0
5.	3	2	2
6.	2	0	0
7.	2	1	0
8.	4	3	3
9.	3	0	1
10.	2	0	0
11	4	1	1
12.	3	1	2
13.	2	1	1
14.	3	1	1
15.	3	2	2
16.	2	0	0

(a): UPDRS stands for *Unified Parkinson's Disease Rating Scale*. (b): The range of *Hoehn and Yahr's Disease Stage* is *1-5*; the range of *UPDRS II-5* and *UPDRS III-18* is *0-4*.

**Table 2:** The descriptive statistics of the speech intelligibility scores and fluency indicators.

Participant no.	Intelligibility score	Repetition	Correction	Omission	Distortion	Unfilled pause	Filled pause	Speaking Rate^a^
1	7	0	0	2	0	1	0	2.66
2	6.5	0	1	0	1	0	0	4.04
3	6.5	0	0	0	0	4	1	2.78
4	7	0	1	0	0	1	0	2.52
5	2.5	1	2	16	1	4	0	1.92
6	7	0	0	0	0	1	0	2.53
7	4	2	1	0	0	11	0	1.96
8	2	0	1	43	3	14	1	1.51
9	6.5	1	0	0	0	6	0	2.57
10	6.5	0	0	0	2	0	0	3.37
11	5	5	1	0	3	16	0	1.91
12	1.5	2	2	19	3	25	3	1.03
13	7	0	0	0	0	0	0	2.85
14	3.5	2	4	0	1	11	0	1.88
15	4	2	1	3	1	34	1	1.31
16	6.5	0	0	0	1	1	0	3.02
Average score	5.19	0.94	0.88	5.19	1	8.06	0.38	2.37
Standard deviation	1.97	1.39	1.09	11.68	1.16	10.02	0.81	0.79

a: Speaking rate was calculated based on the average number of syllables produced by the speaker per second.

**Table 3:** Model summary of the multiple regression.

No.	Model	*R*	*R^2^*	Adjusted *R^2^*	*R^2^* change	*p*
1.	**Severity level**	.628	.395	.352	.395	.009
2.	**Severity level & seven fluency indicators**	.967	.935	.860	.540	.006

**Table 4:** Contributions of severity level and each of the fluency indicators.

Variable	Unstandardized *β*	Standardized *β*	*t*	*p*
Constant	5.640		2.641	.033
Severity level	.638	.232	1.261	.248
Unfilled pause	-.031	-.158	-.737	.485
Filled pause	-.300	-.123	-.744	.481
Speaking rate	-.063	-.025	-.091	.930
Repetition	-.592	-.416	-1.470	.185
Correction	-.687	-.379	-3.249	**.014**
Omission	-.131	-.778	-2.741	**.029**
Distortion	.224	.131	.585	.577

## Data Availability

The data presented in this study are available at the corpus Mobile Device Voice Recordings at King's College London (MDVR-KCL) from both early and advanced Parkinson's disease patients and healthy controls [38] via the link: 10.5281/zenodo.2867216.
